# Enhancing risk communication for climate-sensitive neglected tropical diseases in Northern Tanzania: Integrating agropastoral community knowledge and health system strategies

**DOI:** 10.1371/journal.pntd.0014527

**Published:** 2026-07-14

**Authors:** Chris G. Buse, Emmanuel Sulle, Epiphania Ngowi, Laurie Kidayi, Michelle Lavrov, Alais Ole Morindat, Kahabi G. Isangula

**Affiliations:** 1 Faculty of Health Sciences, Simon Fraser University, Burnaby, Canada; 2 Arusha Climate and Environmental Research Centre, Aga Khan University, Arusha, United Republic of Tanzania; 3 Global South Studies Centre, University of Cologne, Cologne, Germany; 4 Faculty of Environment, Simon Fraser University, Burnaby, Canada; 5 Institute for Poverty, Land and Agrarian Studies, University of the Western Cape, Cape Town, South Africa; 6 Arkaria Impact Center, Monduli, United Republic of Tanzania; 7 School of Nursing and Midwifery, Aga Khan University, Dar Es Salaam, United Republic of Tanzania; University of Calgary, CANADA

## Abstract

Tanzania has made significant progress in reducing number of neglected tropical diseases (NTDs) by using education initiatives and mass drug administration (MDA). However, as East Africa’s climate continues to change, the extant NTD response face challenges as vector distribution and abundance changes in relation patterns of precipitation and increasing temperatures. This research aimed to understand community needs in NTD risk communication and how these needs align with strategies deployed by practitioners and regional health administrators in two districts in northern Tanzania: Arusha, a predominantly urban setting, and Monduli, a primarily rural and agro-pastoral community. The research team conducted an environmental scan of climate-sensitive NTD risk communication materials of relevance to the study area; five focus group discussions that engaged 44 participants from peri-urban and rural communities; and semi-structured interviews (N = 13) with doctors (N = 3), nurses (N = 3), veterinarians (N = 2) and health administrators (N = 5) in English and Swahili. This contribution identifies community preferences in receiving health risk communication materials, practitioner strategies for sharing information about NTDs, and discusses opportunities for action to marry these priorities in the context of the region’s changing climate. Findings suggest key tensions in community needs versus information being provided, and considerable resource disparities and needs between urban and rural populations.

## Author summary

Tanzania has taken important steps and celebrated successes in NTD reduction. Climate change will alter the distribution and incidence of most, if not all, NTDs of relevance to Tanzania. In this study, we compare the existing NTD risk communication strategy as espoused by health system practitioners with the wants and needs of underserved pastoral communities in peri-urban and rural settings. In building on identified tensions, we discuss five opportunities for enhancing the NTD response under a changing climate including: bolstering data utilization and surveillance of NTDs; addressing vaccine hesitancy and misinformation; strategic investment in practitioner training and WaSH programs; enhancing risk communication materials and access to those materials by shifting the communicative burden off of children; and further incorporating traditional knowledge in NTD risk promotion and treatment.

## 1. Introduction

Over the past several years, progress has been made on reducing neglected tropical diseases (NTDs) in the United Republic of Tanzania (hereafter, ‘Tanzania’). However, many of these diseases remain ‘neglected’ (i.e., lacking funding and attention), especially in more rural and remote parts of the world with limited health sector capacity [1]. Moreover, climate change is anticipated to modify the range and prevalence of many infections and NTDs, including diarrheal diseases [2], raising questions for existing strategies for NTD reduction and elimination. To date, Tanzania has made significant progress in reducing NTDs through mass drug administration (MDA) of prophylactic medication to school-aged children, in combination with health education campaigns and water, sanitation and hygiene programs (WaSH) [3].

While Tanzania has celebrated many successes through the development of these initiatives, there is concern nationally and globally that program implementation may not be equitably distributed across regions or populations [4]. In Tanzania, there are noted disparities in access to NTD reduction programs between urban and rural regions [5–7] and according to socioeconomic status [8,9], but particularly for agro-pastoral communities such as the Maasai [10]. As the current Tanzanian strategy for NTD reduction and elimination comes to an end in 2026, there are opportunities to reflect on the success of past initiatives, and chart a course for continued action, particularly related to how communities understand NTDs, and how risk is communicated and addressed by health practitioners [11].

In this contribution, we share insights from a community-engaged research project on the health and risk communication of climate-sensitive NTDs. Semantically, we treat health and risk communication as overlapping and commensurate approaches aimed at rapidly informing the public of certain health risks so that they can take protective or preventative measures. Specifically, this research asks, “how do agro-pastoral community members and health system practitioners understand NTD threats and responses under a changing climate?”. This research question is animated by several interrelated research objectives, including [1] to engage peri-urban and rural community members from Arusha and Monduli in dialogue about NTD risks, and whether climate change plays a role in influencing those perceptions; [2] evaluate community perceptions of how health practitioners are meeting the challenges posed by NTDs in their communities; and [3] documenting health system actions on NTD risk mitigation, and whether climate change is being meaningfully incorporated into practice-based innovations. In doing so, we offer several recommendations to further enhance the national strategy for NTD elimination and reduction.

### 1.1. Priority NTDs and climate change in the United Republic of Tanzania

In Tanzania, the principal efforts aimed at reducing NTDs are through preventive mass drug administration [3]. While the government has enhanced WaSH program delivery, community engagement activities, and health and risk communication campaigns exist, these programs may not reach rural and lower-income residents who struggle with access to clean water, seasonal climate impacts, and a lower understanding of treatment and prevention strategies.

In the northern districts of Tanzania, several NTDs have been highlighted as being a priority for infection control [3], including Schistosomiasis—which has been well-studied both regionally and national [12–15], lymphatic filariasis [16,17], Trachoma [18], soil-transmitted helminths (STH) [13,19–21], and onchocerciasis [22,23]. However, past research has suggested that other NTDs are also endemic and of concern for northern Tanzania, including, but not limited to: rabies [24,25]; leptospirosis [26]; dengue, chikungunya and Rift Valley Fever viruses [27–29]; human fascioliasis [30]; brucellosis [31]; and cystic echinococcosis [32], among others.

Despite progress on studying NTDs globally and regionally in northern Tanzania, there are concerns that climate change will continue to alter the range and prevalence of vectors, and when in combination with other patterns of land-use change, urbanization, population growth and migration, NTD distribution risks rapid change without on-going intervention [33,34]. East Africa in particular is experiencing changes in the distribution of severity of NTDs, primarily driven by changing temperature and precipitation patterns [35].

For example, warmer temperatures may improve motility and accelerated development at higher temperatures for some soil-transmitted helminths, increasing the overall population and risk to humans as temperatures continue to warm [36]; heavy rain and flood events, especially at high latitudes in cool upland areas around with natural or artificial waterways and reservoirs may create conditions for enhanced schistosomiasis risk [37]; habitat suitability for vectors spreading Human African Trypanosomiasis (HAT) may shrink under warming conditions, but consequently force HAT vectors to higher altitudes [38]; tick-borne disease range is being influenced by cross-border movement of livestock, while also supporting more heat-tolerant tick species [39]; mosquitos competent at spreading Rift Valley Fever are anticipated to enhance concentration in the northwest, and continue to spread further south [40]; and the range and risk of lymphatic filariasis infection are predicted to expand, particularly in endemic regions [41]. These changes raise significant challenges for the ongoing treatment of NTDs, but particularly where surgical care is required in already under-resourced settings where climates may experience abrupt or slow onset changes with considerable implications for pathogen development and spread [42].

### 1.2. State of community-engaged knowledge on NTDs and climate change in Northern Tanzania

The current state of knowledge suggests that action on NTDs should be integrated with water, sanitation and hygiene (WaSH) services, immunization programs, vector control strategies, and primary healthcare [43]. Community engagement that has at its core, a strong understanding of place-based contexts to tailor health and risk communication is also essential for furthering reductions in disease onset and ongoing transmission [44]. The research that has been done typically engages with aspects of zoonoses and infectious disease, with limited consideration of climate. For example, many pastoralists understand the possibility of local syndromic zoonotic transmission, but generally tolerate these risks and harms as a reality of their culture and livelihoods [45]. In rural regions, access to reliable health services (including transport, bed nets and related resources) and sanitation are commonly reported challenges, primarily driven by financial cost, and traditional medicines to treat disease are common practice [46]. Additionally, there are variations in knowledge and understanding of certain NTDs among health practitioners [47].

Further evidence reinforces the notion that rural and remote communities may not be benefiting equitably from NTD control programs. In the case of Trachoma control, the SAFE (surgery, antibiotics, facial cleanliness, environmental treatment) strategy has been in place in Tanzania for years, but due to community mistrust of various organizations, poor disease knowledge, environmental factors and traditional lifestyles, many agro-pastoral and low-income communities may not benefiting from these programs [48].

## 2. Methods

### 2.1. Ethics statement

This study received ethical clearance from the research ethics boards of Simon Fraser University (Canada - 30002328), Aga Khan University (Tanzania - AKU/2024/22/fb/03/116), the National Institute for Medical Research (NIMR, Tanzania - NIMR/HQ/R.8a/Vol.IX/4628), and the Tanzania Commission for Science and Technology (COSTECH, Tanzania - CST00000549-2024-2024-00984). Formal consent to participate in the study was obtained from all individuals who agreed to take part based on participant preference. Written consent was obtained from most participants, while verbal consent was obtained from participants who were unable to read or write. Informed consent forms were shared with all participants outlining their role as a research participant, indicating that any recorded information would not be attributable to them as an individual, and that transcripts would be de-identified to maintain confidentiality. Because of the unique context of the focus groups, we followed similar protocols but noted we could not guarantee confidentiality because of the group setting. Data access was restricted only to team members who are the authors of this manuscript.

To address the stated research objectives, this project adopted a qualitative, multi-method comparative case study methodology drawing on key informant interviews and focus group discussions across one peri-urban (southwestern Arusha) and one rural (Monduli) district in northern Tanzania. Monduli and Arusha districts were selected because they are geographically adjacent and offer a comparative context for examining NTD experiences across rural and peri-urban settings. Furthermore, the districts face persistent challenges in the provision of social services such as water supply and health services. These districts were also chosen because NTD risk in both settings remains insufficiently characterized, with limited published prevalence data and to the authors’ knowledge, no previous studies examining community and health practitioner perceptions of NTD risk related to climate change.

### 2.2. Recruitment and data collection

The study employed a purposive referral sampling strategy to capture diverse perspectives of both community members and practitioners involved in the transmission, prevention, response to, and treatment of NTDs. The sampling frame was designed to capture perspectives across multiple administrative and operational levels-reginal, district, and community-in Monduli and Arusha districts. Participants included health professionals and community members at each level.

Sample size was determined interactively during data collection and was guided by the principle of thematic saturation, defined as the point at which no new substantive themes emerged from successive interviews or focus groups (Kerr et al. 2010). This approach is consistent with qualitative research standards prioritizing methodological rigor and transparency over predetermined sample size targets.

Recruitment of community members were led by the Aga Khan University’s (AKU) Arusha Climate and Environmental Research Center (ACER) that is physically situated in a peri-urban part of southwestern Arusha, embedded in pastoral communities where it has forged relationships with community leaders. Outreach officers shared recruitment materials with community leaders through a short briefing note on project objectives and word-of-mouth, encouraging them to pass the information on to interested members of their communities. This approach had the benefit of leveraging existing relationships and using word-of-mouth to invite previously unengaged community members to participate in this study. In Monduli, participants were recruited by the Akaria Impact Center, a local not-for-profit organization aimed at improving the livelihoods of Maasai populations in the face of environmental and social change, which was a key partner on this project, with existing relationships to AKU-ACER. Recruitment was primarily conducted through word of mouth for focus groups. Health system practitioners were recruited by targeted invitation through telephone and email to key contacts working on NTDs region and at regional health centres, hospitals, and veterinary clinics. Additional participants were sourced utilizing referral sampling methods. All participants were eligible to participate if they were aged 18 years or older at the time of participating, were members of an agro-pastoral community in peri-urban Arusha or Monduli—or a health system practitioner in either district—and were willing to provide written or verbal consent (their preference) to have their contributions recorded and utilized for the purposes of research.

Interviews and focus group discussions were conducted in-person in Arusha and Monduli, between May to July 2024. Participants were provided with information about the study in a short briefing note that described its purpose, potential risks, confidentiality, and the benefits. Participants were also informed about how their information will be used and limits of confidentiality. They were advised that confidentiality could not be fully guaranteed due to the group setting; however, they were encouraged to respect each participant’s privacy and opinions and to refrain from sharing any information discussed out of the group. Participants were also discouraged from using any identifiers such as full names, and instead codes or pseudonyms were used throughout the data collection, transcription and analysis. Those who agreed to be recorded were provided with written informed consent prior to participating, and the investigator explained how the recordings would be securely stored and who would have access to them. Audio files, transcripts, and consent forms were stored separately in password-protected devices and secure folders accessible only to the research team. Any identifying information was removed during transcription, and quotations used in reports were anonymized to prevent identification of individual participants. Participants were also informed that they could decline to answer any question, request that the recorder be paused, or withdraw from the discussion at any time without penalty.

All interviews and focus group discussions were conducted in Swahili language to ensure comprehension, unless participants confirmed their willingness to proceed in English. For participants not fluent in Swahili, trained translator facilitated communication in the local language, especially Maasai language. Sessions were held in private to ensure confidentiality and lasted approximately 30–60 minutes. Participants received compensation for their time and travel expenses – a minimum of TZS 15,000 per person plus actual transportation costs.

FGDs with community members sought to learn how their households access clean water and tend to livestock, what their main health concerns are, how climate affects their health, how they seek and obtain health information, and what ways they prefer to receive information generally. Interviews sought to understand what NTD training is given to health practitioners, which NTDs are treated in their locality, what media are the risks of NTDs and other health concerns communicated to the public, how seasonality affects the health of the populations they treat, and what are the existing strategies for NTD reduction and elimination at the regional and district levels. Interviewees were also asked to provide recommendations on engagement with local communities to enhance the health and risk communication of NTDs. Focus groups and interviews were led until data saturation was achieved, while striving to maintain a roughly equal distribution of perspectives from our two case study locations. We considered both geographic and meaning saturation in determining when to stop recruitment, wherein we opted to finalize data collection once interviews and FGDs yielded diminishing new insights [49–52].

All interviews were audio recorded and transcribed. Interviews conducted in English were transcribed using OtterAI software, and interviews conducted in Swahili were transcribed using NottaAI software which was also utilized as a first pass to translate these transcripts into English. We then employed a transcript checking process to verify the accuracy of this translation where a minimum of two Swahili-speaking members of our research team (EN, ES, AM) read the newly translated transcripts against the initial recording to ensure the original intent was maintained in the transcription, and to maximize semantic accuracy and interpretive validity.

### 2.3. Analysis procedures

Transcripts were coded using Dedoose software by three members of the research team, with each transcript being reviewed by at least two team members. We also followed established protocols for thematic analysis of qualitative data [53,54]. Codes were developed iteratively using a combination of inductive and deductive coding strategies. Our team developed an initial coding strategy based on key resources, including the WHO NTD strategy (2021–2030), Tanzania’s NTD Master Plan (2021–2026), and relevant literature, with deductive codes capturing aspects of risk transmission, existing programming, and opportunities for action. These codes primarily reflected the structure of core questions from the interview guide.

We then utilized field note observations and an inductive coding strategy that enabled the data to speak from the context in which it was derived by analyzing data in the context of participant roles and geography to preserve contextual integrity. Each transcript was first read in its entirety in relation to field notes taken by team members immediately after interviews and focus groups. Then, we utilized textual readings of transcripts to develop initial lists of codes around key aspects of how climate change was understood in relation to NTDs, how households were receiving supports (i.e., risk communication, mass drug administration, education campaigns, community health worker visits, etc.) to understand and mitigate NTD harms, and recommendations for enhancing the delivery of these services moving forward. Excerpts were coded according to topics of focus, including a particular health condition (e.g., trachoma, schistosomiasis, amebiasis), practitioner strategies for NTD reduction, strategies for patient education, barriers to health service access or utilization, climate change and seasonality (e.g., floods, droughts), infrastructure (e.g., water, sanitation, and hygiene infrastructure). This approach led to a coding strategy that allowed for new themes to emerge from the data through iterative discussion among our team. We approached our data comparatively, contrasting the experiences of rural and peri-urban focus group participants, as well as perspectives from communities with those of health practitioners to develop a ‘value-added’ approach to reconciling similarities and differences across these contexts [55].

To ensure inter-coder reliability, a minimum of two researchers coded and reviewed the existing coding of all transcripts and field notes. Team members engaged in multiple rounds of coding using and initial sample of five transcripts to develop a code book and to develop familiarity with the analytic framework. In cases of coding discrepancy, consensus was achieved through discussion with the wider team, and in rare cases of disagreement, a two-thirds majority decision-making process was utilized to support code application. Given this project is exploratory and interpretive, we purposefully do not report on quantitative metrics of inter-rater reliability as it is incompatible with this methodology. However, our process of code building and application followed established protocols for code application while building rigour and consistency in coding approaches [56].

### 2.4. Researcher positionality

The research team consisted of three professors—two from Tanzania (ES, KI) and one from Canada (CB), two graduate students (ML, LK), one research contractor (EP) and one agropastoral community leader (AM). Five team members were fluent in Swahili, and all members of the study team were fluent in English. Power dynamics were consciously acknowledged and addressed throughout the data gathering and interpretation process by the authors. Team members acknowledged their positionality is shaped by their social roles, professional status, and prior relationships within the community which had the potential to influence participant responses and team interpretation of data.

Recognizing this, the team approached data collection with an emphasis on reflexivity, transparency, and relational accountability. During data collection, efforts were made to mitigate hierarchical dynamics by adopting participatory and dialogic methods that emphasize mutual respect and co-learning. In the interpretation phase, power dynamics were addressed through collaborative analysis and reflexive conversations among team members. The team critically examined how their own assumptions, and affiliations and community loyalties might shape the interpretations of the data. Interpretive decisions were discussed collectively but defaulted towards centering participant voices. Therefore, power was treated not as something that could be fully eliminated, but as a dynamic force that required ongoing negotiations. The inclusion of an agro-pastoral community member in the team sought to further contextualize our analysis and writing of this manuscript and served as a guiding aspect to ground our insights in the lived realities of agro-pastoral community members in Northern Tanzania.

### 2.5. Limitations

We recognize that while the purposive and referral sampling strategy was appropriate for achieving thematic saturation, it may have limited the representativeness of the sample. Participants were selected based on relevance and accessibility rather than to capture the full diversity of perspectives in Monduli and peri-urban Arusha. Recruitment through established relationships with community leaders and partner organizations may also have introduced selection bias and influenced participation. In addition, despite the use of reflexive and participatory approaches, interviewer characteristics, including personality and perceived authority, may have shaped participants’ responses and contributed to social desirability bias. Finally, the qualitative scope of the study limits the generalizability of findings beyond the two study sites. While the results provide in depth, context specific insights into NTD risk perceptions and practices across rural and peri urban settings, the small sample size and limited geography may limit generalizability and are intended to inform understanding in similar contexts rather than broader inference.

## 3. Results

In total, 57 people were engaged across five focus groups and 13 interviews (see Tables 1 and 2). We now report on key findings before introducing implications for action: [1] the orientation participants had towards climate change as related to NTD transmission risk and its communication; [2] strategies that community members would benefit from and health officials utilize to communicate about NTD transmission in peri-urban Arusha and rural Monduli, and [3] key recommendations emerging at the interface of those strategies that were identified as holding potential for further NTD elimination and reduction moving forward.

**Table 1:** Demographic data of participants in focus groups.

Focus Group		#1- Peri-urban (N = 5)	#2 – Peri-Urban (N = 6)	#3 – Rural (N = 20)	#4 – Peri-urban (N = 6)	#5 – Rural (N = 7)
Variable		Number of Participants				
**Gender**	Male	5	0	7	3	3
	Female	0	6	13	3	4
**Age**	18–29	2	0		0	2
	30–39	0	3		2	0
	40–49	1	2		3	2
	50–59	0	0		1	1
	60+	2	1		0	2
	Unspecified^+^	0	0	20	0	0
**Education**	Primary school	2	5	0	5	0
	Secondary school	3	1	0	1	0
	Unspecified^+^	0	0	20	0	7
**Occupation**	Farmer	2	1	0	1	0
	Pastoralist	0	0	0	0	7
	Entrepreneur	2	1	0	4	0
	Business farming	1	4	0	1	0
	Unspecified^+^	0	0	20	0	0

+ Unspecified category: participants chose not to disclose their information, though most participants in Focus Group 3 are farmers and/or pastoralists.

**Table 2:** Demographic data of interview participants.

Interview	Position	Gender	District	Facility	Age	Education	Years of Experience
1	Nurse	F	Arusha Urban	Public Referral hospital	52	Not reported	30+
2	Doctor	F	Arusha Urban	Private clinic	28	Not reported	1
3	Nurse (retired)	F	Monduli	Dispensary	65	Diploma	36
4	Veterinary Doctor	M	Monduli	Ward	34	Diploma	10
5	Veterinary Doctor	M	Arusha Rural	District Livestock Department	59	Degree	34
6	Medical Doctor	M	Monduli	District Hospital	35	Degree	4
7	Health Official	M	Monduli	District Council	52	Masters	30
8	Health Official	M	Regional		38	Masters	10
9	Health Official	M	Arusha Rural	District Council	Not reported	Diploma	Not reported
10	Health Official	M	Arusha Rural	District Council	33	Diploma	Not reported
11	Health Official	F	Monduli	District Council	58	Diploma	8
12	Nurse	F	Monduli	District Hospital	46	Degree	Not reported
13	Medical Doctor	M	Arusha Rural	District Hospital	35	Degree	10

### 3.1. Climate change and NTD transmission

In terms of NTD and other health concerns raised, there was broad agreement among practitioners and community members on priority health conditions, although not all those raised during interviews were identified NTDs as defined by the Tanzanian NTD Strategy or the World Health Organization. The diseases that are classified by the WHO as NTDs include: Schistosomiasis, Soil-transmitted helminth, Lymphatic filariasis, Trachoma, Rabies, and Onchocerciasis are priority NTDs for the region. Additionally, some NTDs present in the region are zoonotic diseases including Rabies, Brucellosis, and Anthrax due to large livestock populations and human-animal interactions. Non-NTD conditions are health conditions that may co-exist in affected communities or share similar social determinants but are not classified by the WHO as NTDs such as Malaria, diarrhoeal diseases such as amoebiasis allergies, chronic conditions, and pneumonia, among others. The researchers were less interested in where confusion around whether communities understood the distinction between NTDs, zoonotic diseases, or those linked strongly to water, sanitation and hygiene challenges (e.g., amoebiasis), and more interested in how health concerns identified as priorities by communities may be modified by a changing climate. In Monduli district, commonly discussed non-NTD conditions included malnutrition, seasonal allergies, diabetes, respiratory illness, and mental health, with trachoma and soil-transmitted helminth infections being most commonly discussed NTDs. In Arusha district, amebiasis, while not an NTD, was often discussed, particularly in relation to seasonal rains. In both districts, brucellosis, rabies, worm-diseases (inclusive of soil-transmitted helminths), typhoid, cholera, campylobacter infection, joint pain and urinary tract infections were commonly mentioned.

Across both interviews and focus groups, climate change was rarely implicated in relation to the spread of NTDs directly. This was often expressed in relation to the more immediate health concerns that seasonal changes presented to NTD transmission relative to the much longer-term challenges posed by climate change. Participants across both districts consistently framed disease risk through seasonal experience rather than climate change per se, describing a recurring dry-rainy season cycle that shaped water access, livelihoods, and exposure to infection. Particularly in rural Monduli, participants flagged that in many cases, surface water was still shared by humans and livestock, and the longer the dry period, the more likely water was to become contaminated over time. In the rainy season, participants noted that amoebiasis was often prevalent, as were a range of diarrheal symptoms from drinking contaminated flood water.

While community members described seasonal disease risk primarily through lived experience such as drought, dust, flooding and water contamination, health practitioners articulated similar patterns in more NTD disease-specific terms, linking dry periods to trachoma and heavy rains to helminths and schistosomiasis.

One veterinarian in particular shared anecdotal information about transmission risk of cystic echinococcosis between animals and humans, flagging the importance of raising education during periods of weather that may create more active transmission risk, but noting that this is not the norm given communities prefer to treat goats and cows rather than dogs However, they incur huge costs when treating individuals where transmission takes place from dogs. He asserted that although some community members receive the education, they choose not to take preventive measures or may not know the correct vaccine dosage and frequency for treating their animals. He stated:

“Regarding education, we provide a lot of education for livestock management, but the challenge lies in applying/using the education they are given. For example, with the dog vaccine, even if you ask around this village, you’ll find that they haven’t vaccinated their dogs, but their goats and cows have been vaccinated. Dogs are not really given priority. However, they feel the impact when they have to bear the cost of treating someone bitten by a dog. For example, with worms, the effect is visible when you visit livestock and see that the cattle and goats have a lot of worms, especially during heavy rains. This year, due to heavy rains, the worm infestation in the grazing areas has been much larger than during dry periods.” (Veterinary Doctor, Monduli).

Thus, seasonality is clearly a key aspect of both community and health system practitioner needs for communication around NTDs, but this information is often lacking in a timely way and faces challenges around clearer risk communication for specific NTDs as they relate to changing weather and communication patterns. In low-resource contexts, this raises questions about the feasibility of tailoring risk-specific information at given times of year for particular conditions, when mass education campaigns are already difficult to deliver at baseline.

### 3.2. NTD health and risk communication strategies

Despite limited consideration of climate change in communicating about NTDs, our research process surfaced preferences from community members which shared similarities and had distinct differences in preference between peri-urban and rural community-members. Often, these were supported by comments from regional health practitioners around the services available. However, in several cases, there were tensions between what community members experienced from health services and what was thought to be offered by the health system workers. Table 3 below summarizes the differing perspectives of community members and health practitioners in Monduli (rural) and Arusha (peri-urban) for understanding of communication strategies related to NTDs.

**Table 3:** Comparison of community and practitioner perspectives of NTD health and risk communication by district.

Health and Risk Communication Dimension	Arusha (Peri-urban) Community Members	Arusha (Peri-urban) Practitioners	Monduli (Rural) Community Members	Monduli (Rural) Practitioners
Understanding of Climate and NTDs	Climate change rarely named; disease linked to rainy/dry seasons (e.g., amoebiasis during rains)	Explicit links between seasonality, climate patterns, and specific NTDs (e.g., trachoma, helminths	Climate change not named; strong experiential awareness of drought, shared water sources, flooding	Aware of climate-sensitive transmission, especially livestock–human interfaces
Priority Health Concerns	Amoebiasis, diarrheal disease, worms, cholera	WHO-defined NTDs plus surveillance priorities	Malnutrition, respiratory illness, trachoma, helminths	NTDs framed alongside poverty, WaSH deficits, livestock health
Preferred Communication Channels	Radio, TV, mobile phones, churches, community meetings	Verbal education at clinics; school-based education	Word-of-mouth, radio, village meetings, churches	Community meetings, mobile clinics, traditional leaders
Role of Children in Communication	Seen as helpful but limited	Central strategy (school-based MDA and education)	Helpful but not sufficient; adults need direct engagement	Relied upon due to resource efficiency
Trust in Health System	Moderate; increases after personal illness experience	Assumed but recognized as fragile	Often low; shaped by irregular engagement and livelihood impacts	Acknowledged as a challenge requiring relationship-building
Views in Vaccination & MDA	Confusion but some acceptance	Core elimination strategy	Hesitancy linked to costs, mistrust, misinformation	Seen as essential but undermined by resistance
WaSH Conditions	Better infrastructure, though uneven	Recognized as key success factor	Limited access; high seasonal variability	Seen as critical but under-resourced
Traditional Leaders	Trusted but less emphasized	Occasionally engaged	Central and highly trusted	Viewed as effective but not formally integrated

Across both districts, preferred communication channels clustered around trusted interpersonal networks (e.g., community leaders, churches, village meetings) and low-cost mass media (radio and newspapers), with access shaped by rural-peri-urban differences in technology. In both districts, messaging and education were stated to be well-received when delivered by trusted community leaders. Churches and village meetings were typically shared as the best channels to spread messages to large groups at once, and participants highlighted that messaging that motivated behaviour change stemmed primarily from an intent to protect their family. Messaging was exceptionally well-received after participants themselves experienced an illness or witnessed someone else with an illness.

A recruiting strategy described by practitioners involved educating school-aged children, who were expected to relay NTD prevention messages to their families and friends. While this approach was presented as a core pillar of NTD communication, its effectiveness depended on household dynamics, caregiver engagement, and the perceived relevance of the information:

“Our first strategy is provision of health education to the community and to the schools’ children. We are going direct to the schools to educate students on how they can prevent the NTD diseases” (Health Official, Arusha).

Participants offered mixed anecdotes of door-to-door campaigns for specific outbreaks, including COVID-19, where general information was shared, but that community town halls were a typically reliable means to share information. Practitioners also shared that vaccination of animals and education on animal husbandry practices were routine among pastoralists, but that this information did not always translate into practice because of the practitioner’s perceiving some communities to have low levels of education, limited resources or limited capacity to make recommendations actionable.

At a district and regional level, most practitioners referred to the national strategy of MDA, which is mostly administered to school aged children. Regional health officials highlighted how community visits from public health workers educate people on NTDs and have recently attempted to incorporate traditional Maasai leaders to emphasize key messages at community meetings. They also highlighted the importance of ongoing investment in proper flush toilets, sanitation programs, and running water in public facilities like schools, utilizing global aid funding to support some community WaSH facilities to be built, and reiterated the importance of radio and public announcements on NTD control.

Collectively, these findings point to a clear disconnect between how NTD risks are experienced at the community level, and how prevention and preparedness are structured within the health system. While both community members and practitioners recognize strong seasonal patterns in disease transmission, these patterns are rarely translated into timely, disease-specific communication or preventive action. Instead, NTD messaging is often reactive, fragmented, and delivered through generalized channels that may not always leverage trusted local leaders. This gap creates both challenges and opportunities: channels in low-resource contexts where mass communication is already constrained, and opportunities to align existing community-preferred communication pathways with seasonal risk windows.

### 3.3. Recommendations for action

The findings of this study suggest that strengthening NTD elimination efforts do not require new policy frameworks, but rather more context-sensitive implementation of existing strategies. By aligning NTD communication with seasonal risk patterns, risk communication from trusted community voices, and ongoing public health and veterinary programs, policy makers and practitioners can enhance the relevance, timeliness, and effectiveness of prevention efforts in both peri-urban and rural settings.

Findings indicate that while climate is not explicitly recognized by communities as a driver of NTD risk, seasonal patterns are strongly understood and shape perception of disease transmission. This suggests that NTD control efforts should anchor climate-related messaging in locally meaningful concepts such as seasonality (e.g., dry vs. rainy periods) rather than the potentially abstract climate change discourse. There is a clear opportunity to strengthen anticipatory, season-specific prevention and preparedness, particularly for trachoma, soil transmitted helminths, schistosomiasis, and water-borne infections, by aligning education, surveillance, and community readiness (e.g., drugs, WaSH interventions) with predictable seasonal risk periods. However, the resource constraints and delivery challenges underscore the need for pragmatic, targeted approaches rather than highly disease-specific messaging.

Effective NTD health and risk communication depends on local context that is informed by available communication channels, trusted messengers and lived experience of illness. The findings highlight the reliance on low technology and interpersonal communication – such as word-of-mouth, radio, village meetings, churches, and schools in rural settings with limited digital access. Reliance on post-treatment education and school-based messaging places a heavy burden on informal knowledge transmission, which may dilute accuracy and consistency. Strengthening NTD control therefore requires proactive, community-wide education approaches that leverage trusted leaders, align with local motivations (particularly family protection), and address gaps between what health systems believe they provide and what communities actually receive and act upon.

In addition to recommendations that respond to core thematic findings, participants identified a number of implicit and explicit recommendations for enhancing NTD elimination and control in Arusha and Monduli districts, including: [1] bolstering data utilization and surveillance of NTDs; [2] addressing vaccine hesitancy and misinformation; [3] strategic investment in practitioner training and WaSH programs; [4] enhancing risk communication materials and access to those materials by shifting the communicative burden off of children; and [5] further incorporating traditional knowledge in NTD risk promotion and treatment.

#### 3.3.1. Recommendation 1: Bolster data collection and community surveillance of NTDs.

First, in terms of data and surveillance, participants affirmed that the Ministry of Health is still in the process of developing harmonized reporting on NTD incidence and prevalence, and that existing data does not adequately capture urban/rural disparities. Also, within the region health disparities exist:

“For example, it’s difficult to tell how many patients have been treated against intestinal worms because when patients come to the hospital, normally, it’s not just because of the worm infestation, they come with other health problems. And then maybe along the way, doctor diagnose intestinal worms. So as far as I know, the Minister of Health is still in collaboration with the regions and districts in the process of developing a harmonized way of reporting this data” (Health Official, Arusha).

Anecdotally, health practitioners highlighted how rural populations who are generally lower-income agro-pastoralists with generally poorer health status than their urban counterparts and lower access to WaSH infrastructure are differentially impacted by NTDs. However, existing reporting tends to be in aggregate, with some evidence of outreach and engagement from clinic-level data, but with little data disaggregation that would enable better resolution on who is at risk of NTD infection according to geographical and socioeconomic factors:

“What I can say is that three quarters of the population, most of them are suffering from these neglected and non-communicable diseases in our society. Yeah, three quarters. During the financial year, we usually go to the remote areas for screening on a quarterly basis” (Medical Doctor, Monduli).

Nevertheless, practitioners did highlight that national surveys have been helpful at tracking the reduction in cases of NTDs, including Trachoma (a priority NTD for the region of study). However, practitioners flagged the need for ongoing investment to ensure timely data collection can capture trends in incidence and prevalence reduction to better quantify the timeliness of intervention options and their efficacy:

“When we started from 2016, the prevalence was very high… But after SAFE strategy, after giving them antibiotic, after sensitization, Environmental sanitation, and also give them antibiotic, the severity is reduced…If the prevalence is under 5%, there is no need to give them drug for all community.” (Health Official, Arusha).

On-going regular data collection and unlocking the power of disaggregated data that can better answer the question of what works, for whom, and in what contexts, will be essential to leveraging data to inform targeted and ongoing interventions into the future. This could also include routinized collection of community knowledge, attitudes/beliefs, risk perceptions and practices/behaviour so that NTD communication strategies can be further refined and contextualized. Pairing it with downscaled climate projections (i.e., to regional ward scales commensurate with epidemiological surveillance and place-based determinants of health, rather than national scales) will undoubtedly be of importance to project changing risk profiles for the most marginalized populations in the region. To that end, enhancing collaboration between the Tanzania Meteorological Agency and health agencies across the country will enable more sophisticated analysis of climate-related phenomena in concert with disease surveillance.

#### 3.3.2. Recommendation 2: Addressing vaccine hesitancy and misinformation.

Second, community participants and clinicians both identified that vaccine hesitancy and the misinformation around treatments from mass drug administration campaigns were hampering the effectiveness of those programs. On the community side, many parents were unsure about the purpose of vaccines being provided in schools, and similarly, found vaccine mandates for animals to be potentially harmful because of the associated costs and livelihood impacts. In both cases, this stems from a lack of trust between community members and the health system, and the need for more regular visits, relationship building, and offering clear communication campaigns and materials in multiple different mediums. As one nurse reflected, even financial compensation to attend information sessions was reflective of untrusting behaviours around vaccination:

“Currently, when you call people, for example, to educate them about health, they often think there’s money involved. So, getting people to come is challenging. We tried gathering people for the NTD project, educating them, and they were paid. We created various teams to oversee it, but people still think you will come again to give [a vaccination], so we are still far from perfect” (Nurse, Monduli).

The core takeaway from these contributions was that misinformation is primarily a product of trusting relationships, and particularly in rural and remote agro-pastoral settings, there may be a requirement for enhancing the visibility and efficacy of NTD (as well as other health promotion interventions) to continue to build trust:

“…but if you want to get there, talk to people. My message is, make sure you are accepted in the community. You’re accepted, and you are their friend, and you will get the root cause.” (Medical Doctor, Arusha).

#### 3.3.3. Recommendation 3: Continue to make strategic investment in practitioner training and WaSH programming.

Third, practitioners highlighted how often NTD training was provided during formative education, through diploma programs and/or medical or veterinary certification processes. We heard less about opportunities for ongoing on-the-job training provided to practitioners, and even less about the incorporation of climate change into medical and health sciences education. In some instances, practitioners had the opportunity to visit rural places to support the roll out of vaccination programs as part of their training, but this was not universal. Enhancing training opportunities to ensure ongoing awareness and education, appropriate diagnosis, and treatment of NTDs, while being mindful that climate change may create pathways for previously non-endemic NTDs to manifest locally, is warranted. As one participant put it: “the government trying a lot to invest a lot in health facilities and staff. But we still need to invest a lot more” (Medical Doctor, Arusha).

Similar investment is also needed in WaSH programming, where community members in particularly highlighted significant differences in the available of seasonal fresh water. The paradox of water (i.e., too much in the rainy season causing flooding, not enough in the dry season during periods of drought) was rarely linked to climate change’s on-going impacts among those interviewed, raising ongoing importance about educational aspects of the importance of incorporating climate change as part of WaSH education. Regional health officials were quick to point to investment in WaSH as a key aspect of NTD response and that government ministries were working in collaboration with global health projects to implement better access to water and sanitation facilities:

“The government has tried so much to improve services in terms of infrastructure, water supply, education, healthcare, facilities, and all these have impact to people. Once they can access water, they can access education and healthcare services…So, this is to say that the reduced prevalence of NTDs in Arusha is not only because of the ongoing interventions, but also other government measures, including improvement of services, infrastructure, education’’ (Health Official, Arusha).

However, the disparity in distribution of these resources between urban, peri-urban and rural places was simultaneously highlighted by many practitioners and community members themselves. While there are stated priorities of bringing running water to rural places, these types of investments are often expensive, and require additional programs to meet NTD elimination and reduction goals at present:

“Yeah, one of them is to construct and make sure all the villages have running water. Another one is to make sure there is a well and defined toilets, sanitation to every family. Another one is to continue health education to the community, but in the schools, we...have a coordinator for these NTDs. So, it is being taught in the primary schools; water, sanitation and others. So, strategically we are putting in the schools and, in the community,” (Hospital Administrator, Monduli).

At the same time, health practitioners also flagged that certain disease remain neglected due to limited budgets and the need to prioritize certain conditions over others:

“Why isn’t the government investing? We reduced the use of drugs. They made an effort and connected…by linking livestock, health, and associate departments, but it still doesn’t reach the people; diseases like [brucellosis] aren’t taken seriously… in terms of healthcare services, I don’t know, should I say the government is trying, but there are diseases that are completely sidelined (Veterinary Doctor, Arusha)

Prioritizing response is obviously incredibly difficult, especially if robust surveillance is limited. However, ongoing underinvestment or limited prioritization risks further reinforcing neglect for these conditions. Systematic, sustained investment in health training, risk communication/education campaigns, and NTD reduction through WaSH programming and basic infrastructure are all equally necessary.

#### 3.3.4. Recommendation 4: Reduce the communication burden of NTDs on children.

Fourth, the strategic focus on mass drug administration and communication materials in schools was central to all our respondents understanding of where the primary focus of NTD elimination and reduction was coming from in the region. There is a considerable focus on educating children, especially children who are patients, and encouraging them to pass along this information to their families and friends:

“One thing I can advise is that they should continue being educated, especially against traditions that are not beneficial to them. For example, when girls are prevented from going to school and get married at a young age. They should study to help even their parents to get out of that situation... Parents don’t know how to read, but when children learn, they try to help their parents too. And they receive education from children as well because once they get educated, they can assist them” (Nurse, Monduli).

While the approach is laudable, it could be criticized for placing an unfair communicative burden of responsibility on children and raises the question as to the degree to which children can be consistently relied upon to share the best evidence on NTD prevention strategies. It also risks creating intergenerational injustices by virtue of ‘double burdening’ a younger generation which to date has been less implicated in causing the problem (i.e., health risks associated with climate change) that they are more vulnerable to than working aged adult. Few clinics and hospitals studied had physical information sheets on NTDs that could act as a communicative aid, and community members flagged having more diagrammatic representations of NTD transmission risks, symptoms and treatments would be helpful. Moreover, such a strategy may risk only benefiting children, and not older generations who may be less likely to have received formal education or have been vaccinated themselves. As a result, NTD practitioners are increasingly finding alternative modes of peer-to-peer education models, and engaging people at village meetings, religious gatherings and local celebrations:

“We also use an approach of working with individual farmers. Some farmers fully understand this approach. They come to us and request a special day for a personal visit. We visit them at their homes, where you’ll meet their friends or a few relatives. You discuss with them everything they need help with, addressing their concerns from A to Z, and then you resolve the issues. Another method for disseminating information is through community meetings and local gatherings, such as village government meetings” (Veterinary doctor, Arusha).

While participants highlighted that this is the gold standard approach, it was difficult to make commitments on any given year that such meetings would be possible: “If there is no budget, we rarely inform them, but they are very, very keen because they usually understand well” (Medical Doctor, Monduli).

#### 3.3.5. Recommendation 5: Incorporate traditional knowledge in NTD communication.

Finally, practitioners and community-members alike highlighted that a core aspect of ongoing relationship-building and trust-building, was to secure better working relationships with village leaders and traditional healers. Participants highlighted engaging traditional healers is an effective mechanism for ‘breaking resistance’ and as an “appropriate entry strategy” primarily related to sharing communication and ensuring “cultural practices are followed, for example, to successfully promote vaccinations. They use their leaders, prominent figures, as well as elders…They are traditional leaders among the Maasai. When you present any matter to the community, they can spread or deliver it effectively (Veterinary Doctor, Monduli).

Respected community members were seen in both cases to be the principle communicative conduits capable of reaching the majority number of people in rural settings. However, to date, traditional healers are not formally integrated into healthcare strategies around NTD prevention and treatment. Relatedly, participants flagged that real communicative and trusting breakthroughs were achieved first and foremost when centring an understanding of the Maasai community more broadly; not only their customs, but “to first study their environment, cultural systems for solving social problems, and then you see which approach you’ll use to convey your message” (Veterinary Doctor, Arusha).

## 4. Discussion and conclusion

This research suggests that additional engagement with the risks posed by climate change is necessary to protect the health of rural, peri-urban, agro-pastoral communities in Northern Tanzania, aligning with past research [45,46]. Based on engagement with health system practitioners and agropastoral community members in peri-urban Arusha and rural Monduli, this study found that the most raised NTD-related concerns in the region are from soil-transmitted helminth (STH) infections, echinococcosis, brucellosis and trachoma. Notably, STH and trachoma are noted priority NTDs in the national strategy and for the regions under investigation in this study, but echinococcosis and brucellosis do not have the same profile in the national strategy raising on-going questions and challenges about how some NTD conditions may remain under-prioritized and ‘neglected’ aspects of disease transmission, particularly in more rural and pastoral communities across Tanzania and much of East Africa.

Moreover, there is currently limited consideration of and concern for how climate change may alter the distribution or severity of NTDs by health practitioners and community members, including those that may not be currently endemic to the region or only experience episodic outbreaks (e.g., Rift Valley Fever). While this may reflect alternative local epistemologies, it may also be the product of servicing more immediate health concerns of local populations and variations in NTD understanding across the region [47]. Indeed, we highlight how ‘seasonality’ was more important for both case study populations than climate change, illustrating the challenges of focusing on a slow-moving emergency with a long-time horizon that climate change poses to NTDs and other priority non-NTD health concerns among community members (i.e., amebiasis, non-communicable diseases). Nonetheless, this should not be considered as a lack of concern about climate change but may reinforce the notion that health system policy needs to be attenuated to this risk now, to prevent future impacts on already differentially impacted populations.

Further, we highlighted several areas requiring on-going and sustained attention and resourcing to enhance the health and risk communication and reduce the burden of NTDs in Arusha and Monduli. To that end, the biggest opportunity is to achieve a more equitable balance in the distribution of resources to rural and peri-urban areas relative to urban contexts in Tanzania which have already seen the largest demonstrable benefits of NTD investment.

Perhaps more importantly, our research highlights the need to give concerted thought to the relationships that are being built between health systems practitioners and more rural and remote communities. This approach holds promise not only for NTD reduction and elimination, but also for promoting the health of populations as they continue to undergo the epidemiological transition to life-style driven diseases implicated for cardiovascular, cerebrovascular, and respiratory health. While this research is supportive of past calls to imagine the deployment of new technologies such as smart phones to support risk communication and mass health education campaigns [57], centring community needs may highlight the limitations of such an approach in areas with low smart phone penetration. Here, a rollout of community-engaged communication campaigns, peer-to-peer communication models, and incorporating traditional healers hold significant promise [58], and our research further reinforces the efficacy of such approaches that has been previously established [59,60].

Clearly, neglected tropical diseases have received the moniker ‘neglected’ because of systematic underinvestment. As Tanzania continue to reap the rewards of investing in NTD reduction and limitation, enhancing focus on the sustained delivery of WaSH programming and targeted communications will be helpful, so long as they are mindful of local context, accessible to all groups of people regardless of their education status, and build sustained trust between communities and health system practitioners. Attending to the well-noted disparities in urban and rural health [5,6,8,9], as well as the provision of health services needs to be at the core of a next generation of thinking if NTDs are to be eliminated in the Tanzanian context, while integrating projections of altered disease transmission risk under a the countries rapidly changing climate.
